# Ethnic Differences in Cardiometabolic Risk Factors for Premature Coronary Artery Disease in Iranian Women: Results From the Iran Premature Coronary Artery Disease (IPAD) Study

**DOI:** 10.7759/cureus.112149

**Published:** 2026-07-06

**Authors:** Abdullah Bhuiyan, Muhammad Afridi, Khadijeh Shamsi, Mina Hmimas, Mohammadamin M Esfahani, Noushin Mohammadifard, Ehsan Zarepur, Nizal Sarrafzadegan

**Affiliations:** 1 Faculty of Health Sciences, Queen's University, Kingston, CAN; 2 Interventional Cardiology Research Center, Isfahan Cardiovascular Research Institute, Isfahan University of Medical Sciences, Isfahan, IRN; 3 Faculty of Medicine and Health Sciences, McGill University, Quebec, CAN

**Keywords:** cardiometabolic risk factors, coronary angiography, ethnicity, iran, premature coronary artery disease, women’s cardiovascular health

## Abstract

Introduction

Premature coronary artery disease (PCAD) is highly prevalent in many low- and middle-income countries, including Iran. Women remain underdiagnosed in clinical practice and underrepresented in PCAD research, particularly across ethnically diverse populations. Iran comprises multiple major ethnic groups with distinct cultural, dietary, and lifestyle characteristics that may differentially shape cardiometabolic risk, providing a compelling rationale for ethnicity-stratified investigation of PCAD in women.

Methods

We analyzed data from the multicenter Iran Premature Coronary Artery Disease (IPAD) case-control study. Cases were women with angiographically confirmed obstructive coronary artery disease occurring at or before 70 years of age, whereas controls had normal coronary arteries on angiography. Although PCAD is commonly defined as CAD occurring before 65 years of age in women, the IPAD study extended the upper age limit to 70 years to reflect the later average age of CAD onset in Iranian women compared with Western populations and to capture a broader at-risk demographic in the context of Iran’s epidemiological profile. This age criterion is consistent with the published IPAD study protocol.

Results

A total of 1,609 Iranian women aged ≤70 years were included, comprising 789 PCAD cases and 820 controls across eight ethnic groups from 15 cities. Across ethnicities, women with PCAD were consistently older than controls and were characterized by lower educational attainment, predominantly low socioeconomic status, very low smoking prevalence, and higher adiposity. Cardiometabolic risk factors varied by ethnicity: hypertension and diabetes were more common among cases in most groups, low high-density lipoprotein cholesterol (HDL-C) levels were highly prevalent in both cases and controls, and family history of cardiovascular disease (CVD) was more frequent among cases in several ethnicities. Ethnicity-stratified analyses revealed distinct risk factor profiles across groups. Among Fars women, hypertension, diabetes, low HDL-C, and family history of CVD were associated with higher odds of PCAD. Among Arab women, hypertension and diabetes were the dominant risk factors. Hypertriglyceridemia was the primary lipid-related predictor among Gilak women, while family history of CVD was the most prominent risk factor among Kurd and Bakhtiari women.

Conclusions

Traditional cardiometabolic risk factors are associated with PCAD among Iranian women, but their magnitudes vary across ethnic groups, supporting ethnicity-informed risk assessment and tailored prevention strategies.

## Introduction

Cardiovascular disease (CVD) is the leading cause of mortality worldwide, responsible for over 17 million deaths annually, with coronary artery disease (CAD) accounting for more than 7 million of these deaths [1]. Premature coronary artery disease (PCAD), defined as CAD occurring before 55 years of age in men and 65 years of age in women, represents approximately 10% of all CAD cases globally and disproportionately affects developing countries [1]. These early-onset cases impose substantial personal and societal burdens by affecting individuals during their most productive years [2]. In Iran, where nearly 40% of deaths are attributable to CVD, PCAD has emerged as a significant public health concern, with prevalence estimates ranging from 2% to 10%, depending on the region [1]. This combination of high overall CAD burden and a considerable proportion of premature cases underscores the importance of studying PCAD in the Iranian population.

Addressing PCAD in women is particularly important. Despite CVD being a leading cause of death in women, a persistent misconception remains that women, especially premenopausal women, are at lower risk for heart disease [3]. This bias has contributed to the under-recognition and undertreatment of CAD in women [4]. Younger women frequently present with atypical symptoms or myocardial ischemia in the absence of obstructive coronary disease, complicating diagnosis [5,6]. In addition, both lower patient awareness and a reduced index of clinical suspicion among physicians have been reported, leading to delayed diagnosis and less aggressive preventive management compared with men [5,7]. These disparities have been associated with poorer outcomes in women, particularly as certain traditional risk factors, including diabetes and dyslipidemia, confer disproportionately higher CAD risk in women [8].

Another critical dimension in Iran is the country’s substantial ethnic diversity and its potential influence on PCAD. Cardiovascular risk factor prevalence and disease burden are not uniform across ethnic groups [9]. Iran comprises multiple major ethnic populations with distinct cultural, dietary, lifestyle, and socioeconomic characteristics that may modify how cardiometabolic risk factors translate into disease [9]. In this study, ethnicity is conceptualized primarily as a social and cultural construct shaped by shared language, geographic origin, dietary traditions, and lifestyle practices, rather than as a proxy for fixed biological or genetic differences. While genetic factors may contribute to observed differences in disease risk across groups, the ethnic variation documented here is interpreted within this broader social and cultural framework. The Iran Premature Coronary Artery Disease (IPAD) study was established to investigate these differences and is among the first large-scale efforts in Asia to examine PCAD across ethnicities [9]. Early IPAD findings demonstrate substantial heterogeneity in PCAD prevalence and risk factor profiles, with some ethnic groups experiencing significantly higher rates of premature CAD even after accounting for conventional risk factors [2].

In summary, PCAD represents a major health issue globally and in Iran, and there is a compelling need to explore its determinants among Iranian women across the country’s diverse ethnic spectrum. International evidence increasingly demonstrates that ethnic background modifies cardiovascular risk in women, showing substantial variation in the prevalence and impact of traditional cardiometabolic risk factors, including diabetes, dyslipidemia, and hypertension, across racial and ethnic groups, and highlighting that behavioral, environmental, and social determinants of health further compound these disparities [10]. Despite this growing body of evidence, women from ethnically diverse low- and middle-income countries remain critically underrepresented in cardiovascular research, and no large-scale study has systematically characterized ethnic differences in cardiometabolic risk factor profiles specifically among Iranian women. Our study addresses this gap directly by leveraging data from the IPAD project to examine ethnic differences in the association between traditional cardiometabolic risk factors, including hypertension, diabetes, dyslipidemia, and family history of CVD, and PCAD among Iranian women. Focusing on women is particularly warranted given that traditional risk factors such as diabetes and dyslipidemia confer disproportionately greater coronary risk in women than in men, that women more frequently present with atypical symptoms leading to delayed diagnosis, and that female-specific biological factors, including hormonal transitions, further complicate risk stratification. Such insights are crucial for developing targeted prevention and intervention strategies, ultimately helping to reduce the burden of premature heart disease in all sectors of Iran’s population.

## Materials and methods

Study design and population

This study is a pre-specified sex-specific analysis of female participants enrolled in the IPAD study, a multicenter case-control investigation initiated in 2020 and conducted across multiple provinces in Iran between 2020 and 2024. The IPAD study was designed to evaluate the prevalence and determinants of PCAD across major Iranian ethnic groups. Details of the overall study design and methodology have been published previously [9].

**Figure 1:**
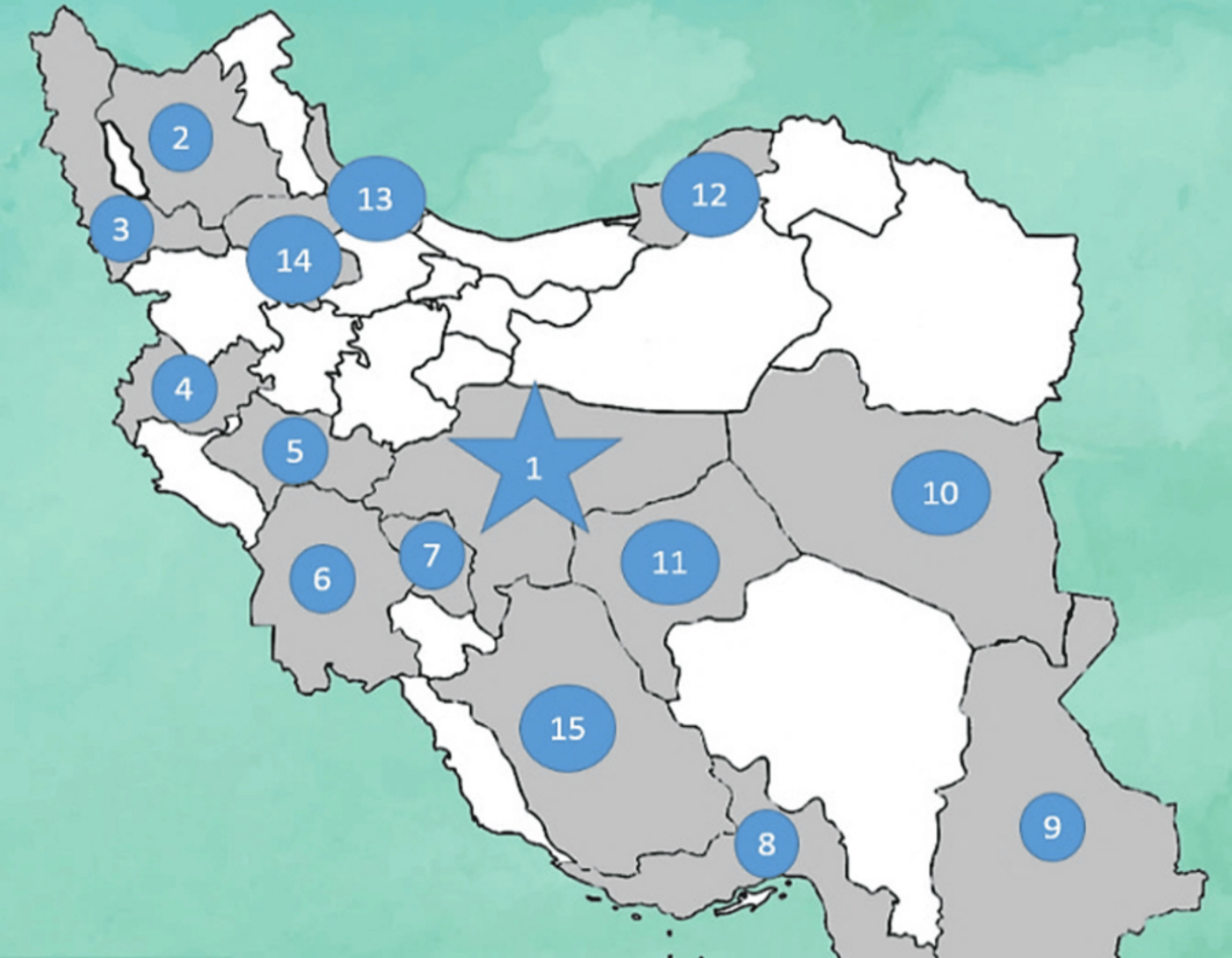
Cities included in the IPAD study recruitment framework across Iran. Participating cities included Isfahan (1), Tabriz and Maragheh (2), Orumiyeh (3), Kermanshah (4), Khorramabad (5), Ahvaz (6), Shahrekord (7), Bandar Abbas (8), Zahedan and Zabol (9), Birjand (10), Yazd (11), Gorgan (12), Rasht (13), Zanjan (14), and Shiraz (15). Adapted from Zarepur E et al. (2020). IPAD: Iran Premature Coronary Artery Disease.

Women who underwent diagnostic coronary angiography at participating hospitals with catheterization laboratories were eligible for inclusion. Participants were recruited from multiple referral centers located in regions representing Iran’s major ethnic populations, including Fars, Azari, Gilak, Kurd, Arab, Lor, Qashqaei, and Bakhtiari ethnicities. Ethnicity was defined based on self-report and confirmation that both parents belonged to the same ethnic group, in accordance with the IPAD protocol. Demographic, clinical, laboratory, and lifestyle data were collected by trained physicians and research staff using standardized questionnaires and protocols. Recruitment centers were distributed across multiple cities in Iran, with each center focusing on one or more predominant local ethnic groups; the geographic distribution of participating cities and recruitment centers has been previously illustrated in a published IPAD map (Figure 1) [11].

Women aged ≤70 years who underwent coronary angiography were eligible for inclusion in this analysis. Although PCAD is conventionally defined as CAD occurring before 65 years of age in women, the IPAD study protocol extended the upper age limit to 70 years based on evidence that Iranian women experience a later average onset of CAD compared with Western populations and to ensure adequate representation of women presenting with premature or early-onset disease across Iran’s diverse ethnic groups. This age criterion was pre-specified in the published IPAD protocol [11] and is applied consistently across all ethnic strata in the present analysis. Women aged 65-70 years were not analyzed as a separate subgroup; however, all regression models were adjusted for age to account for the age distribution within and across ethnic groups, mitigating the potential for age-related confounding. PCAD cases were defined as women with angiographically confirmed coronary artery disease, characterized by ≥75% stenosis in at least one major epicardial coronary artery or ≥50% stenosis of the left main coronary artery.

Control participants were women with normal coronary arteries on angiography and no evidence of obstructive coronary disease. It is acknowledged that both cases and controls were recruited from women undergoing clinically indicated coronary angiography rather than from the general population. As such, controls likely had symptoms or risk factors sufficient to prompt angiographic investigation, which may not be representative of truly healthy community-based individuals. This design introduces the potential for Berkson bias, whereby the estimated associations between cardiometabolic risk factors and PCAD may differ from those that would be observed in a population-based study. This limitation is discussed further in the Limitations section.

Women with a prior history of CAD, including previous coronary artery bypass grafting (CABG), percutaneous coronary intervention (PCI), or balloon angioplasty, were excluded. Participants with incomplete demographic, ethnicity, or angiographic data were also excluded. After applying the inclusion and exclusion criteria, a total of 1,609 women were included in the final analysis, comprising 789 PCAD cases and 820 controls.

Data collection and measurements

Standardized questionnaires were used to collect information on demographic characteristics, socioeconomic status (SES), educational attainment, ethnicity, lifestyle behaviors, and family history of CVD [9]. Anthropometric measurements, including height, weight, waist circumference, and BMI, were obtained using standardized protocols.

Blood samples were collected after a minimum 12-hour fast and analyzed at centralized laboratories. Cardiometabolic risk factors assessed in this study included hypertension, diabetes mellitus, dyslipidemia, and family history of CVD. Hypertension was defined as self-reported physician diagnosis, current use of antihypertensive medications, or documented blood pressure ≥140/90 mmHg. Diabetes mellitus was defined by self-reported physician diagnosis or use of glucose-lowering medications.

Dyslipidemia was evaluated using standard lipid thresholds, including hypertriglyceridemia, defined as triglycerides ≥150 mg/dL; hypercholesterolemia, defined as total cholesterol ≥240 mg/dL; high low-density lipoprotein cholesterol, defined as LDL-C ≥160 mg/dL; and low high-density lipoprotein cholesterol, defined as HDL-C <50 mg/dL. Obesity was defined as BMI ≥30 kg/m². Family history of CVD was defined as a reported history of CAD or related cardiovascular conditions in first-degree relatives. Further details on the study methods are described in depth in the IPAD methodology section [9,11].

Statistical analysis

In this study, all analyses were conducted in two sections, descriptive and analytical, on 1,609 individuals categorized into eight ethnic groups. Continuous data across different groups were described as means ± SDs, while categorical data were presented as frequencies and percentages. Differences between case and control groups were assessed according to the type of variable using the Chi-square test or Fisher’s exact test across ethnicities. Logistic regression analysis was used to obtain ORs and 95% CIs for the association between PCAD as the dependent variable and cardiometabolic risk factors, including hypertension, diabetes mellitus, hypertriglyceridemia, hypercholesterolemia, high LDL-C, low HDL-C, and family history of CVD. The first model was adjusted for age, and the subsequent model was additionally adjusted for SES (low, high). Additional covariates, including BMI, waist circumference, smoking status, educational attainment, and physical activity, were considered for inclusion in multivariable models; however, given the very low prevalence of smoking across all ethnic strata, the high collinearity between educational attainment and SES, and the wide variance and inconsistent case-control patterning of physical activity and adiposity measures across groups, these variables were not included as additional adjusters in the primary models to avoid model instability in smaller ethnic strata. These variables are described later in the Results section, and their distributions are interpreted in the context of the results. Formal interaction testing between ethnicity and individual cardiometabolic risk factors was not performed; instead, ethnicity-stratified regression models were fitted separately for each ethnic group to examine differences in the direction and magnitude of associations across strata. No corrections for multiple comparisons were applied, given the exploratory nature of these analyses; therefore, the findings should be interpreted as hypothesis-generating rather than confirmatory, and replication in independent cohorts is warranted. Data analysis was conducted using SPSS version 27. A p-value <0.05 was considered statistically significant.

## Results

Table 1 summarizes the basic characteristics and behavioral risk factors across the eight ethnic groups. Across ethnicities, the largest ethnic stratum was Fars women (PCAD n=314; control n=421), followed by Azari (PCAD n=137; control n=96) and Kurd (PCAD n=91; control n=121) women. Other ethnic groups, including Gilak, Bakhtiari, Arab, Lor, and Qashqaei women, were represented in smaller numbers, respectively.

**Table 1:** Basic characteristics and behavioral risk factors in women aged ≤70 years with and without PCAD, stratified by ethnicity (IPAD Study, Iran, 2020-present). ᵃ PCAD cases were defined as women with ≥75% stenosis in at least one major coronary artery or ≥50% stenosis of the left main coronary artery on angiography. Controls had normal coronary arteries. All participants were aged ≤70 years. ᵇ Proportions represent each ethnic group’s share of all PCAD cases (n=789) or all controls (n=820) in the total study sample. ᶜ Values in parentheses represent proportions within each ethnic PCAD or control group, not proportions of the total sample. ᵈ Physical activity was measured in MET-minutes per week. MET refers to the metabolic equivalent of task. Higher values indicate greater activity levels. Physical activity was assessed using the International Physical Activity Questionnaire (IPAQ), with total MET-minutes per week calculated by summing occupational, household, transport, and leisure activity domains without truncation of extreme values. ᵉ Smoking status categories may not sum to the ethnic group total in all strata due to missing responses from a subset of participants. Proportions are calculated among participants with available smoking data. ᶠ Education category counts may not sum to the ethnic group total in all strata due to missing data for a small number of participants. Proportions are calculated among participants with available education data. Continuous variables, including age, BMI, and waist circumference, are presented as mean ± SD. Note: Results for Lor controls (n=15), Qashqaei PCAD cases (n=24), and Arab women (PCAD n=43; control n=37) should be interpreted with caution due to small sample sizes. PCAD: Premature coronary artery disease; WC: Waist circumference; MET: Metabolic equivalent of task; MET-minutes/week: Metabolic equivalent of task minutes per week; IPAD: Iran Premature Coronary Artery Disease study.

Characteristics	Ethnicities															
	Fars PCAD	Fars Control	Azari PCAD	Azari Control	Gilak PCAD	Gilak Control	Kurd PCAD	Kurd Control	Arab PCAD	Arab Control	Lor PCAD	Lor Control	Qashqaei PCAD	Qashqaei Control	Bakhtiari PCAD	Bakhtiari Control
n (proportion of total PCAD participants or total control participants)ᵃᵇ	314 (0.398)	421 (0.513)	137 (0.174)	96 (0.117)	88 (0.112)	43 (0.052)	91 (0.115)	121 (0.148)	43 (0.055)	37 (0.045)	60 (0.076)	15 (0.018)	24 (0.030)	38 (0.046)	32 (0.041)	49 (0.060)
Age (years)	58.49 ± 6.97	54.59 ± 7.23	59.98 ± 7.96	55.66 ± 8.29	60.28 ± 5.98	54.33 ± 9.40	58.70 ± 6.50	54.12 ± 7.81	57.26 ± 7.82	51.24 ± 7.75	60.83 ± 8.12	54.53 ± 9.70	59.17 ± 7.12	54.42 ± 5.76	57.47 ± 8.21	53.94 ± 6.62
Education, n (%)ᶜᶠ																
Illiterate and elementary	232 (0.74)	295 (0.70)	105 (0.77)	66 (0.69)	69 (0.78)	23 (0.53)	77 (0.85)	83 (0.69)	24 (0.56)	23 (0.62)	49 (0.83)	12 (0.80)	20 (0.83)	34 (0.89)	27 (0.84)	44 (0.90)
Secondary and high school	72 (0.23)	104 (0.25)	28 (0.20)	24 (0.25)	19 (0.22)	19 (0.44)	9 (0.10)	31 (0.26)	18 (0.42)	12 (0.32)	10 (0.17)	3 (0.20)	3 (0.13)	3 (0.08)	4 (0.13)	5 (0.10)
University	10 (0.03)	22 (0.05)	4 (0.03)	6 (0.06)	0 (0.00)	1 (0.02)	5 (0.05)	7 (0.06)	1 (0.02)	2 (0.05)	0 (0.00)	0 (0.00)	1 (0.04)	1 (0.03)	1 (0.03)	0 (0.00)
Socioeconomic status score, n (%)ᶜ																
Low	200 (0.64)	271 (0.64)	93 (0.68)	52 (0.54)	73 (0.83)	29 (0.67)	69 (0.76)	86 (0.71)	25 (0.58)	15 (0.41)	42 (0.70)	14 (0.93)	21 (0.88)	29 (0.76)	29 (0.91)	38 (0.78)
High	114 (0.36)	150 (0.36)	44 (0.32)	44 (0.46)	15 (0.17)	14 (0.33)	22 (0.24)	35 (0.29)	18 (0.42)	22 (0.59)	18 (0.30)	1 (0.07)	3 (0.13)	9 (0.24)	3 (0.09)	11 (0.22)
Physical activity (MET-minutes/week)ᵈ	36261.98 ± 34063.87	37931.83 ± 34482.33	32160.90 ± 36831.55	38985.20 ± 41777.74	31763.06 ± 43221.10	14848.56 ± 22286.61	27713.09 ± 31258.07	36818.87 ± 40824.05	24961.79 ± 29426.89	30130.83 ± 27514.02	39603.37 ± 41402.73	54218.07 ± 41179.13	45108.08 ± 47997.15	49138.26 ± 40326.70	42356.66 ± 53875.33	54750.43 ± 50055.90
Smoking, n (%)ᶜ^ᵉ^																
Current smoker	7 (0.02)	7 (0.02)	3 (0.02)	4 (0.04)	1 (0.01)	0 (0.00)	2 (0.02)	5 (0.04)	1 (0.02)	1 (0.03)	4 (0.07)	2 (0.13)	3 (0.13)	0 (0.00)	2 (0.06)	2 (0.04)
Ex-smoker	15 (0.05)	12 (0.03)	1 (0.01)	2 (0.02)	2 (0.02)	0 (0.00)	4 (0.04)	5 (0.04)	0 (0.00)	0 (0.00)	6 (0.10)	3 (0.20)	2 (0.08)	4 (0.11)	2 (0.06)	0 (0.00)
Never smoker	292 (0.93)	402 (0.95)	67 (0.49)	42 (0.44)	85 (0.97)	43 (1.00)	84 (0.92)	110 (0.91)	33 (0.77)	22 (0.59)	50 (0.83)	10 (0.67)	19 (0.79)	34 (0.89)	28 (0.88)	47 (0.96)
BMI (kg/m²)	29.46 ± 4.96	29.44 ± 4.95	30.37 ± 5.39	30.78 ± 5.08	28.56 ± 5.36	31.00 ± 5.50	28.99 ± 5.14	30.34 ± 5.92	27.80 ± 4.04	28.59 ± 4.70	28.33 ± 4.04	31.91 ± 4.48	30.23 ± 4.84	29.47 ± 4.36	28.87 ± 4.50	30.18 ± 4.81
WC (cm)	100.97 ± 11.91	98.03 ± 11.74	104.42 ± 17.27	107.71 ± 15.90	95.09 ± 16.82	104.77 ± 15.06	96.66 ± 12.45	97.53 ± 13.54	101.93 ± 14.04	99.41 ± 12.25	96.63 ± 15.55	102.07 ± 7.41	101.04 ± 9.74	98.75 ± 10.51	101.03 ± 11.28	100.65 ± 12.26

Other ethnic groups, including Gilak, Bakhtiari, Arab, Lor, and Qashqaei women, were represented in smaller numbers (Table 1). Within all ethnicities, women with PCAD were older than controls (e.g., Fars: 58.5 ± 7.0 vs 54.6 ± 7.2 years; Azari: 60.0 ± 8.0 vs 55.7 ± 8.3 years; Gilak: 60.3 ± 6.0 vs 54.3 ± 9.4 years; Kurd: 58.7 ± 6.5 vs 54.1 ± 7.8 years), underscoring age as a key gradient between groups.

Educational attainment skewed heavily toward illiterate/elementary levels across all groups, with university education uncommon. Overall, SES was predominantly low across cases and controls, although the proportion of low SES also varied by ethnicity (e.g., Azari cases 93 (68%) vs controls 52 (54%); Lor controls 14 (93%)). Physical activity showed wide dispersion and clear between-ethnicity heterogeneity (e.g., Fars ≈36-38k MET-minutes/week; Azari ≈32-39k MET-minutes/week; Lor controls ≈54k MET-minutes/week), rather than a consistent case-control pattern. Among participants with available smoking data, smoking prevalence was uniformly low: most were never-smokers, and current smoking was rare in all strata. Adiposity was elevated overall (BMI ~28-31 kg/m²; waist circumference ~96-108 cm), with modest and directionally inconsistent case-control differences across ethnicities.

Table 2 demonstrates the prevalence of cardiometabolic risk factors, stratified by ethnicity. In general, cases had a higher prevalence than controls for several factors, although magnitudes varied by group. Hypertension was more frequent among cases in most ethnicities (Fars 189 (60%) vs 188 (45%); Kurd 44 (48%) vs 44 (36%); Arab 27 (63%) vs 9 (24%)), with smaller differences or near-parity in others (e.g., Azari 89 (65%) vs 59 (61%)). Diabetes tended to be more common among cases across multiple groups (Fars 155 (49%) vs 111 (26%); Azari 62 (45%) vs 32 (33%); Gilak 52 (59%) vs 15 (36%); Arab 23 (53%) vs 11 (30%); Bakhtiari 15 (47%) vs 15 (31%)), with narrower gaps in some strata (e.g., Kurd 32 (35%) vs 30 (25%); Qashqaei 9 (38%) vs 8 (21%)).

**Table 2:** Distribution of cardiometabolic risk factors among women with and without PCAD, stratified by ethnicity. Data are presented as number (proportion). A p-value <0.05 was considered statistically significant. ᵃ Calculated using the Chi-square test. ᵇ Calculated using Fisher’s exact test. HDL-C: High-density lipoprotein cholesterol; LDL-C: Low-density lipoprotein cholesterol; CVD: Cardiovascular disease; PCAD: Premature coronary artery disease.

Characteristics, n (proportion within PCAD/control group)	Ethnic groups																							
	Fars PCAD	Fars Control	Fars p-value	Azari PCAD	Azari Control	Azari p-value	Gilak PCAD	Gilak Control	Gilak p-value	Kurd PCAD	Kurd Control	Kurd p-value	Arab PCAD	Arab Control	Arab p-value	Lor PCAD	Lor Control	Lor p-value	Qashqaei PCAD	Qashqaei Control	Qashqaei p-value	Bakhtiari PCAD	Bakhtiari Control	Bakhtiari p-value
Hypertension	189 (0.60)	188 (0.45)	<0.001ᵃ	89 (0.65)	59 (0.61)	0.584ᵃ	52 (0.59)	25 (0.58)	0.917ᵃ	44 (0.48)	44 (0.36)	0.08ᵃ	27 (0.63)	9 (0.24)	<0.001ᵃ	46 (0.77)	10 (0.67)	0.51ᵇ	16 (0.67)	18 (0.47)	0.137ᵇ	15 (0.47)	21 (0.43)	0.722ᵇ
Diabetes mellitus	155 (0.49)	111 (0.26)	<0.001ᵃ	62 (0.45)	32 (0.33)	0.068ᵃ	52 (0.59)	15 (0.36)	0.013ᵃ	32 (0.35)	30 (0.25)	0.1ᵃ	23 (0.53)	11 (0.30)	0.032ᵃ	30 (0.50)	6 (0.40)	0.488ᵇ	9 (0.38)	8 (0.21)	0.157ᵇ	15 (0.47)	15 (0.31)	0.138ᵃ
Hypertriglyceridemia	204 (0.65)	227 (0.54)	0.003ᵃ	54 (0.39)	41 (0.43)	0.615ᵃ	72 (0.82)	27 (0.63)	0.017ᵃ	57 (0.63)	65 (0.54)	0.193ᵃ	15 (0.35)	11 (0.30)	0.624ᵃ	38 (0.63)	9 (0.60)	0.811ᵇ	15 (0.63)	19 (0.50)	0.335ᵇ	20 (0.63)	33 (0.67)	0.654ᵃ
Hypercholesterolemia	152 (0.48)	171 (0.41)	0.035ᵃ	45 (0.33)	36 (0.38)	0.463ᵃ	59 (0.67)	23 (0.53)	0.132ᵃ	36 (0.40)	32 (0.26)	0.043ᵃ	15 (0.35)	11 (0.30)	0.624ᵃ	30 (0.50)	5 (0.33)	0.247ᵇ	12 (0.50)	12 (0.32)	0.147ᵇ	13 (0.41)	17 (0.35)	0.589ᵃ
High LDL-C	148 (0.47)	159 (0.38)	0.011ᵃ	44 (0.32)	37 (0.39)	0.311ᵃ	58 (0.66)	24 (0.57)	0.333ᵃ	34 (0.37)	33 (0.27)	0.118ᵃ	15 (0.35)	11 (0.30)	0.624ᵃ	29 (0.48)	3 (0.20)	0.047ᵇ	12 (0.50)	11 (0.29)	0.095ᵇ	13 (0.41)	18 (0.37)	0.725ᵃ
Low HDL-C	265 (0.84)	306 (0.73)	<0.001ᵃ	124 (0.91)	92 (0.96)	0.124ᵃ	76 (0.86)	39 (0.91)	0.477ᵃ	72 (0.79)	86 (0.71)	0.183ᵃ	43 (1.00)	36 (0.97)	0.462ᵇ	53 (0.88)	12 (0.80)	0.408ᵇ	20 (0.83)	27 (0.71)	0.271ᵃ	30 (0.94)	43 (0.88)	0.469ᵇ
Family history of CVD	194 (0.62)	213 (0.51)	0.003ᵃ	62 (0.45)	38 (0.40)	0.389ᵃ	34 (0.39)	19 (0.44)	0.543ᵃ	65 (0.71)	68 (0.56)	0.023ᵃ	25 (0.58)	22 (0.59)	0.905ᵃ	45 (0.75)	9 (0.60)	0.335ᵇ	15 (0.63)	20 (0.53)	0.445ᵃ	17 (0.53)	16 (0.33)	0.067ᵇ

Patterns for dyslipidemia were heterogeneous: case proportions for hypertriglyceridemia and hypercholesterolemia were often higher in Fars, Gilak, Kurd, and several smaller groups, but not uniformly so; high LDL-C differences were mixed by ethnicity. Low HDL-C was extremely common across the entire sample, affecting roughly 70-90% of women in many ethnicities (e.g., 265 (84%) of Fars cases vs 306 (73%) of controls). As it was highly prevalent in both PCAD and non-PCAD groups, this widespread dyslipidemia likely reduced the observable difference between cases and controls, making its effect appear weaker in some ethnicities despite its biological importance. Family history of CVD was commonly more frequent among cases (e.g., Fars 194 (62%) vs 213 (51%); Kurd 65 (71%) vs 68 (56%); Lor 45 (75%) vs 9 (60%); Bakhtiari 17 (53%) vs 16 (33%)). Table 3 presents ORs and 95% CIs for cardiometabolic risk factors and PCAD across ethnicity-stratified logistic regression models. Crude, Model 1 (adjusted for age), and Model 2 (adjusted for age and SES) were fitted separately within each ethnic group; differences in the direction and magnitude of associations across strata were observed but should be interpreted descriptively, as no formal interaction testing or heterogeneity analysis was performed. In Fars women, several cardiometabolic risk factors showed significant crude associations with higher odds of PCAD, including hypertension, diabetes, low HDL-C, hypertriglyceridemia, hypercholesterolemia, high LDL-C, and family history of CVD. After adjusting for age and socioeconomic status, four factors remained significantly associated with increased PCAD risk: hypertension (OR=1.54; 95% CI: 1.13-2.10), diabetes (OR=2.48; 95% CI: 1.80-3.41), low HDL-C (OR=2.12; 95% CI: 1.44-3.11), and family history of CVD (OR=1.74; 95% CI: 1.27-2.38). In contrast, the associations for hypertriglyceridemia, hypercholesterolemia, and high LDL-C were no longer statistically significant after adjustment, indicating that these lipid measures were attenuated once age and SES were controlled for.

**Table 3:** Odds ratios and 95% confidence intervals for cardiometabolic risk factors and PCAD in ethnicity-stratified logistic regression models among Iranian women. Data are presented as ORs with 95% confidence intervals and were obtained from logistic regression. A p-value <0.05 was considered statistically significant. Crude: unadjusted. Model 1: adjusted for age. Model 2: adjusted for age and socioeconomic status. - indicates that the odds ratio for low HDL-C among Arab women could not be estimated due to complete or quasi-complete separation, whereby the near-universal frequency of low HDL-C in both case and control groups (100% of cases and 97% of controls) left insufficient exposure variability for model estimation. PCAD: Premature coronary artery disease; LDL-C: Low-density lipoprotein cholesterol; HDL-C: High-density lipoprotein cholesterol; CVD: Cardiovascular disease.

Characteristics	Model	Fars OR (95% CI)	Fars p-value	Azari OR (95% CI)	Azari p-value	Gilak OR (95% CI)	Gilak p-value	Kurd OR (95% CI)	Kurd p-value	Arab OR (95% CI)	Arab p-value	Lor OR (95% CI)	Lor p-value	Qashqaei OR (95% CI)	Qashqaei p-value	Bakhtiari OR (95% CI)	Bakhtiari p-value
Hypertension	Crude	1.87 (1.39, 2.52)	<0.001	1.16 (0.68, 2.00)	0.584	1.04 (0.50, 2.18)	0.917	1.64 (0.94, 2.85)	0.08	5.25 (1.98, 13.89)	<0.001	1.64 (0.48, 5.61)	0.429	2.22 (0.77, 6.42)	0.14	1.18 (0.48, 2.88)	0.722
Hypertension	Model 1	1.54 (1.13, 2.09)	0.007	0.94 (0.53, 1.66)	0.829	0.76 (0.34, 1.72)	0.509	1.42 (0.80, 2.54)	0.235	3.83 (1.38, 10.64)	0.01	1.16 (0.31, 4.29)	0.823	1.49 (0.47, 4.67)	0.498	0.82 (0.31, 2.18)	0.692
Hypertension	Model 2	1.54 (1.13, 2.10)	0.006	0.97 (0.55, 1.73)	0.931	0.70 (0.31, 1.61)	0.407	1.42 (0.79, 2.54)	0.238	3.93 (1.39, 11.11)	0.01	0.81 (0.18, 3.60)	0.78	1.54 (0.48, 4.88)	0.467	0.80 (0.30, 2.15)	0.656
Diabetes mellitus	Crude	2.72 (2.00, 3.71)	<0.001	1.65 (0.96, 2.84)	0.069	2.60 (1.21, 5.56)	0.014	1.65 (0.91, 2.99)	0.102	2.72 (1.08, 6.86)	0.034	1.50 (0.47, 4.74)	0.49	2.25 (0.72, 7.01)	0.162	2.00 (0.79, 5.03)	0.141
Diabetes mellitus	Model 1	2.48 (1.80, 3.41)	<0.001	1.54 (0.88, 2.70)	0.127	2.29 (1.01, 5.19)	0.047	1.46 (0.78, 2.72)	0.239	2.88 (1.06, 7.80)	0.038	1.57 (0.47, 5.24)	0.465	2.16 (0.65, 7.19)	0.211	1.53 (0.57, 4.07)	0.399
Diabetes mellitus	Model 2	2.48 (1.80, 3.41)	<0.001	1.55 (0.88, 2.72)	0.126	2.18 (0.95, 4.97)	0.065	1.45 (0.78, 2.71)	0.242	2.91 (1.06, 7.98)	0.038	1.45 (0.41, 5.14)	0.566	2.14 (0.64, 7.15)	0.216	1.59 (0.59, 4.30)	0.36
Hypertriglyceridemia	Crude	1.58 (1.17, 2.14)	0.003	0.87 (0.51, 1.48)	0.615	2.67 (1.17, 6.07)	0.019	1.44 (0.83, 2.52)	0.194	1.27 (0.49, 3.25)	0.624	1.15 (0.36, 3.67)	0.811	1.67 (0.59, 4.73)	0.337	0.81 (0.32, 2.05)	0.654
Hypertriglyceridemia	Model 1	1.33 (0.97, 1.82)	0.074	0.78 (0.45, 1.35)	0.377	2.38 (0.98, 5.74)	0.054	1.27 (0.71, 2.27)	0.421	0.75 (0.26, 2.14)	0.592	0.84 (0.24, 2.95)	0.783	1.16 (0.38, 3.59)	0.795	0.63 (0.23, 1.71)	0.367
Hypertriglyceridemia	Model 2	1.34 (0.98, 1.83)	0.069	0.76 (0.44, 1.33)	0.335	2.55 (1.04, 6.21)	0.04	1.27 (0.71, 2.27)	0.423	0.78 (0.27, 2.29)	0.657	0.89 (0.23, 3.35)	0.859	1.21 (0.39, 3.78)	0.741	0.67 (0.25, 1.83)	0.437
Hypercholesterolemia	Crude	1.37 (1.02, 1.84)	0.036	0.82 (0.47, 1.41)	0.463	1.77 (0.84, 3.73)	0.134	1.82 (1.02, 3.26)	0.044	1.27 (0.49, 3.25)	0.624	2.00 (0.61, 6.55)	0.252	2.17 (0.76, 6.21)	0.15	1.29 (0.51, 3.23)	0.589
Hypercholesterolemia	Model 1	1.14 (0.84, 1.55)	0.4	0.71 (0.40, 1.26)	0.241	1.30 (0.58, 2.92)	0.53	1.63 (0.89, 3.01)	0.116	0.75 (0.26, 2.14)	0.592	1.59 (0.46, 5.52)	0.464	1.52 (0.49, 4.70)	0.467	1.02 (0.39, 2.67)	0.973
Hypercholesterolemia	Model 2	1.15 (0.84, 1.56)	0.387	0.70 (0.40, 1.24)	0.226	1.31 (0.58, 2.96)	0.515	1.62 (0.88, 3.00)	0.121	0.78 (0.27, 2.29)	0.657	1.82 (0.49, 6.70)	0.368	1.60 (0.51, 5.01)	0.42	1.09 (0.41, 2.91)	0.86
High LDL-C	Crude	1.47 (1.09, 1.98)	0.011	0.75 (0.44, 1.30)	0.311	1.45 (0.68, 3.08)	0.334	1.59 (0.89, 2.85)	0.119	1.27 (0.49, 3.25)	0.624	3.74 (0.96, 14.62)	0.058	2.45 (0.85, 7.11)	0.098	1.18 (0.47, 2.94)	0.725
High LDL-C	Model 1	1.25 (0.92, 1.70)	0.159	0.65 (0.36, 1.14)	0.134	1.14 (0.51, 2.57)	0.745	1.37 (0.74, 2.52)	0.32	0.75 (0.26, 2.14)	0.592	3.09 (0.76, 12.55)	0.114	1.68 (0.54, 5.25)	0.375	0.93 (0.35, 2.42)	0.878
High LDL-C	Model 2	1.25 (0.92, 1.71)	0.153	0.64 (0.36, 1.13)	0.124	1.15 (0.51, 2.59)	0.741	1.36 (0.73, 2.51)	0.334	0.78 (0.27, 2.29)	0.657	3.40 (0.80, 14.56)	0.098	1.71 (0.54, 5.38)	0.359	0.98 (0.37, 2.60)	0.975
Low HDL-C	Crude	2.03 (1.40, 2.95)	<0.001	0.41 (0.13, 1.31)	0.134	0.65 (0.20, 2.15)	0.479	1.54 (0.81, 2.93)	0.185	-	1	1.89 (0.43, 8.40)	0.401	2.04 (0.57, 7.34)	0.277	2.09 (0.40, 11.08)	0.385
Low HDL-C	Model 1	2.10 (1.43, 3.09)	<0.001	0.44 (0.14, 1.44)	0.175	0.82 (0.23, 2.87)	0.751	1.43 (0.73, 2.78)	0.296	-	1	1.71 (0.36, 8.05)	0.495	1.90 (0.50, 7.19)	0.347	1.49 (0.27, 8.35)	0.652
Low HDL-C	Model 2	2.12 (1.44, 3.11)	<0.001	0.43 (0.13, 1.42)	0.167	0.82 (0.23, 2.93)	0.76	1.42 (0.73, 2.77)	0.307	-	1	1.58 (0.32, 7.74)	0.571	2.21 (0.56, 8.69)	0.256	1.48 (0.26, 8.48)	0.659
Family history of CVD	Crude	1.58 (1.17, 2.13)	0.003	1.26 (0.74, 2.14)	0.39	0.80 (0.38, 1.67)	0.544	1.95 (1.09, 3.48)	0.024	0.95 (0.39, 2.31)	0.905	2.00 (0.61, 6.55)	0.252	1.50 (0.53, 4.26)	0.446	2.34 (0.94, 5.84)	0.069
Family history of CVD	Model 1	1.74 (1.28, 2.38)	<0.001	1.33 (0.77, 2.30)	0.312	0.78 (0.35, 1.74)	0.548	2.03 (1.10, 3.74)	0.023	1.33 (0.50, 3.55)	0.565	1.76 (0.51, 6.09)	0.372	1.23 (0.40, 3.77)	0.713	3.20 (1.17, 8.73)	0.023
Family history of CVD	Model 2	1.74 (1.27, 2.38)	<0.001	1.31 (0.76, 2.28)	0.336	0.78 (0.35, 1.74)	0.545	2.15 (1.15, 4.02)	0.017	1.49 (0.54, 4.13)	0.439	1.81 (0.49, 6.70)	0.376	1.29 (0.42, 3.98)	0.661	3.17 (1.15, 8.74)	0.026

Among Azari women, all cardiometabolic risk factors showed non-significant associations with PCAD in both the crude and adjusted models. Hypertension, diabetes, hypertriglyceridemia, hypercholesterolemia, and high LDL-C all demonstrated attenuation with adjustment, although all p-values remained non-significant. The confidence intervals were not wide, reflecting the relatively large sample size of this ethnic group. Overall, the main change after adjustment was the reduction in effect sizes, although all p-values remained non-significant.

In Gilak women, most cardiometabolic risk factor associations were reduced after adjustment, but only hypertriglyceridemia remained significant in Model 2 (OR=2.55, 95% CI: 1.04-6.21). This association is statistically borderline, with a confidence interval that only marginally excludes the null, and may not withstand correction for multiple testing; it should therefore be interpreted with caution and considered hypothesis-generating rather than definitive. Diabetes was significant in Model 1 but attenuated in Model 2 (OR=2.18, 95% CI: 0.95-4.97), suggesting that the association between diabetes and PCAD in Gilak women may be partially mediated or confounded by socioeconomic factors.

In Kurd women, family history of CVD was significantly associated with higher odds of PCAD (OR=2.15, 95% CI: 1.15-4.02), whereas other factors were not significant after adjustment.

Arab women demonstrated adjusted associations for hypertension (OR=3.93, 95% CI: 1.39-11.11) and diabetes (OR=2.91, 95% CI: 1.06-7.98); however, these estimates should be interpreted with considerable caution given the small sample size of this stratum (43 cases, 37 controls), which produced wide confidence intervals reflecting substantial uncertainty and limited precision. Other lipid endpoints were non-significant. Low HDL-C could not be estimated in the logistic regression model for Arab women due to complete or quasi-complete separation, whereby all or nearly all Arab women in both the case and control groups had low HDL-C, leaving insufficient variability in the exposure to estimate an odds ratio.

In both Lor and Qashqaei women, in the fully adjusted model, only hypertension and hypertriglyceridemia showed associations with PCAD, although these associations were not significant. In Lor women, hypertension (OR=0.81, 95% CI: 0.18-3.60) and hypertriglyceridemia (OR=0.89, 95% CI: 0.23-3.35) suggested a potential inverse trend. In contrast, among Qashqaei women, although the adjusted ORs for the same variables decreased compared with the crude model, they remained associated with increased odds of PCAD: hypertension (OR=1.54, 95% CI: 0.48-4.88) and hypertriglyceridemia (OR=1.21, 95% CI: 0.39-3.78). Other adjusted associations were non-significant, reflecting small strata and wide intervals.

Finally, in Bakhtiari women, family history of CVD was significantly associated with higher odds of PCAD (OR=3.17, 95% CI: 1.15-8.74), while other risk factors did not reach significance after adjustment. Together, these findings highlight distinct ethnicity-specific profiles: multifactor signals in Fars women; hypertension and diabetes in Arab women; triglycerides in Gilak women; and family history signals in Kurd and Bakhtiari women, with several smaller strata limited by precision.

## Discussion

In this multi-ethnic study of Iranian women, traditional cardiometabolic risk factors, including hypertension, diabetes, low HDL-C, hypertriglyceridemia, hypercholesterolemia, and family history of CVD, were associated with PCAD. However, the observed direction and magnitude of these associations differed across ethnic strata in the stratified models, although formal interaction testing was not performed and these differences should be interpreted descriptively rather than as statistically confirmed ethnic heterogeneity. These findings are broadly consistent with international evidence. The landmark INTERHEART case-control study, conducted across 52 countries, demonstrated that nine modifiable risk factors, including dyslipidemia, hypertension, and diabetes, account for over 90% of the population-attributable risk of myocardial infarction in both men and women and across all major ethnic groups, while also showing that the relative importance of individual risk factors varied by region and population [12]. A sex-specific analysis of INTERHEART further showed that hypertension and diabetes conferred disproportionately greater risk of myocardial infarction in women than in men across diverse ethnic groups [13], consistent with the prominent role of these factors observed among Fars and Arab women in the present study. Studies from other Middle Eastern populations similarly report high burdens of diabetes, dyslipidemia, and hypertension among women with coronary artery disease, with diabetes emerging as a particularly strong sex-specific risk factor [14]. Among South Asian populations, where premature CAD burden is disproportionately high, dyslipidemia and insulin resistance have been identified as dominant risk factors, paralleling the lipid-related findings observed in Gilak women in this cohort [15]. Taken together, these international comparisons suggest that the cardiometabolic risk factor patterns observed in Iranian women are broadly aligned with global evidence, while the observed between-group differences in their relative importance highlight the value of ethnicity-stratified analyses.

Ethnicity-stratified models revealed distinct patterns across groups: hypertension and diabetes were prominent predictors in Fars and Arab women, hypertriglyceridemia was the only statistically significant lipid-related predictor in Gilak women in the fully adjusted model, and family history of CVD was the most prominent risk factor among Kurd and Bakhtiari women. These observations suggest that although classic cardiometabolic risk factors remain central to PCAD, their observed associations with disease differ across ethnic groups in this dataset; however, because no formal tests of effect modification by ethnicity were performed, these patterns should be considered exploratory and hypothesis-generating rather than evidence of confirmed differential effects.

Several cross-ethnic patterns help contextualize these associations. Age differences between cases and controls were observed across all groups. Low HDL-C was extremely common across all ethnicities, affecting approximately 70-100% of both cases and controls. This finding, while striking, is consistent with previously reported population-level data from Iran, where low HDL-C has been identified as the most prevalent lipid abnormality, particularly among women, and has been attributed to a combination of dietary patterns high in refined carbohydrates, physical inactivity, and high rates of metabolic syndrome in this population. The near-universal frequency of low HDL-C across both cases and controls likely attenuated the observable case-control difference for this risk factor in several ethnic strata, despite its established biological importance in coronary artery disease. Regarding laboratory consistency, blood samples were analyzed at centralized laboratories using standardized protocols, as described in the IPAD methodology, supporting the reliability and comparability of lipid measurements across the 15 recruitment centers. Physical activity also showed wide between-group dispersion; however, as noted in the Methods and Limitations sections, these values reflect IPAQ-derived estimates without truncation and should be interpreted with caution rather than as reliable indicators of true between-ethnicity differences in activity level. The clustering of family history of CVD in certain ethnic groups may reflect shared household and environmental exposures in addition to any hereditary component and should not be interpreted as evidence of purely inherited susceptibility.

Our results align with evidence that diabetes and dyslipidemia have strong effects on coronary disease in women [16-19]. They also reflect prior IPAD findings, which identified elevated frequencies of PCAD cases among certain ethnic groups, including Arabs, Azaris, and Gilaks, in the overall IPAD cohort [2]; however, it is important to note that the present case-control design does not permit estimation of population-level disease risk or prevalence, and the observed case distributions across ethnic strata reflect the study’s sampling framework rather than true ethnic differences in PCAD occurrence. Decision-tree analyses in the IPAD cohort identified ethnicity-specific predictors, such as fasting blood sugar in Gilaks, opium use and hypertension in Lors, and older age and male sex in Kurds [20]. While opium use was not measured in the current analysis, these prior findings provide useful context for interpreting the ethnicity-specific patterns observed here. Regional studies similarly report high frequencies of obesity, dyslipidemia, diabetes, and hypertension among individuals with premature CAD [21].

The observed between-group differences in risk factor associations likely reflect a combination of behavioral, environmental, and socioeconomic influences, although the present study did not directly measure most of these factors, and the following explanations should be understood as hypotheses derived from prior literature rather than conclusions supported by the current data. Prior literature has noted that consanguinity in some communities, such as Arab communities, may contribute to disease clustering, although this likely reflects a combination of shared household, dietary, and environmental exposures, as well as possible hereditary factors, rather than purely genetic susceptibility, and no measures of consanguinity or genetic relatedness were collected in this study [2]. High rates of obesity and metabolic syndrome in Gilak women have been linked in prior studies to cultural dietary patterns and lifestyle transitions [2,22], although dietary patterns were not assessed in the present analysis. It has also been suggested that extreme heat in southern provinces may limit physical activity among Arab women [23] and that higher rates of opium use in Lor communities may influence cardiovascular risk [20,22]; however, neither environmental conditions nor opium use were evaluated in the current study, and these explanations remain speculative in the present context.

These findings have important clinical implications. Given the associations of hypertension and diabetes with PCAD, particularly among Fars and Arab women, and the association of hypertriglyceridemia with PCAD among Gilak women, early screening and culturally adapted prevention programs targeting these specific risk factors may provide significant benefit in these groups [24]. Atherogenic dyslipidemia, which was common across this cohort, may require proactive management through lifestyle modification and medical therapy [25-27]. Research indicates that women are often undertreated for cardiovascular risk due to the misconception that they are at lower risk at younger ages [5]. Our findings suggest that clinicians should consider intervening earlier in women with established cardiometabolic risk factors, particularly those with a strong family history of CVD, although the present case-control design does not permit identification of specific ethnic groups as having higher population-level disease risk.

Incorporating ethnicity into clinical risk assessment may improve the identification of women at elevated risk, since commonly used tools such as the Framingham and ASCVD calculators do not account for Iran’s ethnic diversity and may misestimate risk for certain groups [28,29]. At the public health level, regions with higher observed frequencies of PCAD cases in prior IPAD analyses, such as Gilan, East Azerbaijan, and Khuzestan, may benefit from targeted screening programs, although population-level risk estimates from dedicated epidemiological studies would be needed to formally prioritize such efforts.

Limitations

This study has several limitations that should be considered when interpreting the findings. First, the case-control design inherently limits causal inference, as the temporal relationship between cardiometabolic risk factors and PCAD cannot be established from cross-sectional exposure data collected at the time of angiography. Additionally, because both cases and controls were recruited from women undergoing clinically indicated coronary angiography rather than from the general population, the study is subject to Berkson bias. Controls likely presented with symptoms or risk factors sufficient to prompt angiographic investigation and may not be representative of truly healthy community-based individuals. This may have elevated the prevalence of cardiometabolic risk factors among controls relative to the general population, potentially attenuating the observed associations between risk factors and PCAD. Future studies incorporating community-based or population-representative control groups would help clarify the magnitude of this bias and provide more generalizable effect estimates.

Second, the inclusion of women up to 70 years of age extends beyond the conventional definition of PCAD in women, which is typically defined as CAD onset before 65 years of age. While this age criterion was pre-specified in the published IPAD protocol and justified by evidence of later average CAD onset in Iranian women relative to Western populations, it may introduce some conceptual imprecision in the application of the PCAD label. Future analyses restricted to women younger than 65 years would allow for a more stringent examination of premature disease in line with conventional definitions.

Third, several ethnic strata, particularly Arab, Lor, and Qashqaei women, had small sample sizes, resulting in unstable logistic regression estimates with wide confidence intervals. Findings from these groups should be considered exploratory and interpreted with considerable caution, as the limited statistical precision precludes definitive conclusions about risk factor associations with PCAD in these populations.

Fourth, no corrections for multiple comparisons were applied across the large number of ethnicity-specific tests conducted. Given the exploratory nature of these analyses, some nominally statistically significant findings may reflect type I error, and all results should be interpreted as hypothesis-generating rather than confirmatory, pending replication in independent cohorts.

Fifth, formal interaction testing between ethnicity and individual cardiometabolic risk factors was not performed. The ethnicity-stratified regression models fitted in this study allow observation of differences in the direction and magnitude of associations across groups but do not constitute a formal test of effect modification by ethnicity. Future studies with larger, adequately powered samples should incorporate likelihood ratio tests or other formal interaction analyses to rigorously evaluate whether the associations between cardiometabolic risk factors and PCAD differ statistically across Iranian ethnic groups.

Sixth, the multivariable models were adjusted only for age and socioeconomic status, and residual confounding from unmeasured variables cannot be excluded. Variables including dietary patterns, detailed medication use, menopausal status, female-specific risk factors such as gestational diabetes and preeclampsia, and psychosocial factors were not collected or incorporated into the analyses and may confound the observed associations. Additionally, opium use, which has been identified as a relevant risk factor in prior IPAD analyses, was not measured in the present study.

Seventh, physical activity estimates should be interpreted with caution, as values were derived from the International Physical Activity Questionnaire (IPAQ) without application of the recommended truncation ceiling. IPAQ is known to overestimate total physical activity, particularly when all domains are summed without truncation and in Middle Eastern population contexts. As such, physical activity data in this study are presented descriptively only and were not incorporated into the multivariable adjustment models.

Finally, this study relied on self-reported family history of CVD. Although family history is often interpreted as an indicator of genetic susceptibility, it may also reflect shared lifestyle patterns, dietary habits, environmental exposures, and cultural practices that were not measured directly. The contribution of hereditary versus environmental factors to the observed family history associations cannot be disentangled from the present data alone. Future studies incorporating genetic markers or detailed household-level environmental data would help clarify the relative contributions of these pathways.

## Conclusions

This multi-ethnic case-control analysis of Iranian women found that traditional cardiometabolic risk factors, including hypertension, diabetes, dyslipidemia, and family history of CVD, were associated with PCAD across ethnic groups. Ethnicity-stratified models revealed differences in the direction and magnitude of these associations across groups; however, as no formal interaction testing was performed, these observed differences should be considered exploratory and hypothesis-generating rather than evidence of confirmed effect modification by ethnicity. These findings suggest that a single uniform risk profile may be insufficient to characterize PCAD risk across Iran’s ethnically diverse female population, although this interpretation requires validation in prospective studies with adequate statistical power and formal interaction analyses.

Given the underrecognition of CVD in women, particularly at younger ages, these results support earlier screening and proactive management of cardiometabolic risk factors among women in diverse populations. Future research integrating genetic, environmental, dietary, and behavioral data in prospective designs will be essential to clarify the mechanisms underlying the observed ethnic differences in cardiometabolic risk factor associations and to inform culturally adapted prevention strategies for women in Iran.

## References

[1] Vahedi M, Taherpour N, Aghajani MH, Pourhoseingholi MA, Pourhoseingholi A (2023). Knowledge, attitude, and practice regarding cardiovascular disease among coronary artery disease and premature coronary artery disease patients referred to Imam Hossein Hospital, Tehran, Iran. J Tehran Heart Cent.

[2] Babahajiani M, Zarepur E, Khosravi A (2023). Ethnic differences in the lifestyle behaviors and premature coronary artery disease: a multi-center study. BMC Cardiovasc Disord.

[3] Ryczkowska K, Adach W, Janikowski K, Banach M, Bielecka-Dabrowa A (2023). Menopause and women's cardiovascular health: is it really an obvious relationship?. Arch Med Sci.

[4] Al Hamid A, Beckett R, Wilson M (2024). Gender bias in diagnosis, prevention, and treatment of cardiovascular diseases: a systematic review. Cureus.

[5] Desai S, Munshi A, Munshi D (2021). Gender bias in cardiovascular disease prevention, detection, and management, with specific reference to coronary artery disease. J Midlife Health.

[6] Joseph NM, Ramamoorthy L, Satheesh S (2021). Atypical manifestations of women presenting with myocardial infarction at tertiary health care center: an analytical study. J Midlife Health.

[7] Burt VL, Whelton P, Roccella EJ (1995). Prevalence of hypertension in the US adult population. Results from the Third National Health and Nutrition Examination Survey, 1988-1991. Hypertension.

[8] Fujita Y, Morimoto T, Tokushige A, Ikeda M, Shimabukuro M, Node K, Ueda S (2022). Women with type 2 diabetes and coronary artery disease have a higher risk of heart failure than men, with a significant gender interaction between heart failure risk and risk factor management: a retrospective registry study. BMJ Open Diabetes Res Care.

[9] Zarepur E, Mohammadifard N, Nedaeinia R (2024). Preliminary results of Iran Premature Coronary Artery Disease (IPAD study) and implementation of IPAD Biobank: a case-control study. Arch Iran Med.

[10] Mehta LS, Velarde GP, Lewey J (2023). Cardiovascular disease risk factors in women: the impact of race and ethnicity: a scientific statement from the American Heart Association. Circulation.

[11] Zarepur E, Mohammadifard N, Mansourian M (2020). Rationale, design, and preliminary results of the Iran-premature coronary artery disease study (I-PAD): a multi-center case-control study of different Iranian ethnicities. ARYA Atheroscler.

[12] Yusuf S, Hawken S, Ôunpuu S (2004). Effect of potentially modifiable risk factors associated with myocardial infarction in 52 countries (the INTERHEART study): case-control study. Lancet.

[13] Gupta K, Junaid V, Qureshi MA (2024). Health data sciences and cardiovascular diseases in South Asia: Innovations and challenges in digital health. Curr Atheroscler Rep.

[14] Sayed AI (2022). Gender differences in coronary artery disease, clinical characteristics, and angiographic features in the Jazan Region, Saudi Arabia. Cureus.

[15] Anand SS, Islam S, Rosengren A (2008). Risk factors for myocardial infarction in women and men: insights from the INTERHEART study. Eur Heart J.

[16] Subramanian G, Muthusamy P, Kaliyamurthy T, Ganesan M (2025). Difference in coronary artery disease in women and men in recent trials. Indian J Cardiovasc Dis Women.

[17] Al-Delaimy WK, Manson JE, Solomon CG, Kawachi I, Stampfer MJ, Willett WC, Hu FB (2002). Smoking and risk of coronary heart disease among women with type 2 diabetes mellitus. Arch Intern Med.

[18] de Jong M, Woodward M, Peters SA (2020). Diabetes, glycated hemoglobin, and the risk of myocardial infarction in women and men: a prospective cohort study of the UK Biobank. Diabetes Care.

[19] Khoja A, Andraweera PH, Lassi ZS (2023). Risk factors for premature coronary heart disease in women compared to men: systematic review and meta-analysis. J Womens Health (Larchmt).

[20] Moezi Bady SA, Salmani F, Zarepur E (2025). Investigating ethnic differences in risk factors and severity of developing premature coronary artery disease: Predicting the effect of risk factors through decision tree analysis in a multicenter case-control study; Results from Iran Premature Coronary Artery Disease (IPAD study). J Cardiovasc Thorac Res.

[21] Al-Khlaiwi T, Habib SS, Bayoumy N, Al-Khliwi H, Meo SA (2024). Identifying risk factors and mortality rate of premature coronary artery disease in young Saudi population. Sci Rep.

[22] Hajian-Tilaki KO, Heidari B (2007). Prevalence of obesity, central obesity and the associated factors in urban population aged 20-70 years, in the north of Iran: a population-based study and regression approach. Obes Rev.

[23] Najafi F, Soltani S, Karami Matin B (2020). Socioeconomic - related inequalities in overweight and obesity: findings from the PERSIAN cohort study. BMC Public Health.

[24] Lagisetty PA, Priyadarshini S, Terrell S, Hamati M, Landgraf J, Chopra V, Heisler M (2017). Culturally targeted strategies for diabetes prevention in minority population. Diabetes Educ.

[25] Mosca L (2005). Management of dyslipidemia in women in the post-hormone therapy era. J Gen Intern Med.

[26] Berisha H, Hattab R, Comi L, Giglione C, Migliaccio S, Magni P (2025). Nutrition and lifestyle interventions in managing dyslipidemia and cardiometabolic risk. Nutrients.

[27] Katsiki N, Nikolic D, Montalto G, Banach M, Mikhailidis DP, Rizzo M (2013). The role of fibrate treatment in dyslipidemia: an overview. Curr Pharm Des.

[28] D'Agostino RB Sr, Pencina MJ, Massaro JM, Coady S (2013). Cardiovascular disease risk assessment: insights from Framingham. Glob Heart.

[29] Wong ND, Budoff MJ, Ferdinand K (2022). Atherosclerotic cardiovascular disease risk assessment: an American Society for Preventive Cardiology clinical practice statement. Am J Prev Cardiol.

